# Genomic determinants in advanced endometrial cancer patients with sustained response to hormonal therapy- case series and review of literature

**DOI:** 10.3389/fonc.2023.1188028

**Published:** 2023-07-03

**Authors:** Divya Chukkalore, Anisha Rajavel, Divya Asti, Meekoo Dhar

**Affiliations:** Department of Hematology/Oncology, Staten Island University Hospital, Staten Island, NY, United States

**Keywords:** endometrial cancer, TCGA, next generation sequencing, hormone therapy, genomics

## Abstract

The incidence of endometrial cancer is increasing, however treatment options for advanced disease are limited. Hormonal therapy has demonstrated positive outcomes for Stage IV EC. Next generation sequencing (NGS) has increased our understanding of molecular mechanisms driving EC. In this case series, we selected six patients at our institution with Stage IV, hormone receptor positive, endometrial cancer currently being treated with hormonal therapy. All patients achieved SD for at least ≥ 1.5 years. We studied NGS data on all six patients to assess for any common genomic marker which could predict the SD of at least 1.5 years achieved in this group. Institutional Review Board (IRB) approval was obtained from Staten Island University Hospital and Northwell Health, New York. PTEN, PIK3CA, PIK3R1, and ARID1A mutations were found in 83%, 67% 50%, and 67% of patients respectively. TP53 and FGFR2 were both found in 50% of patients. All patients were positive for estrogen and/or progesterone receptor (ER+ and/or PR+). We did not find any one common mutation that could have predicted the observed response (or SD of ≥1.5 years) to hormone therapy. However, our data reflects the prevalence of various mutations reported in literature: (1) Hormone Receptor status is a positive prognostic indicator (2) PTEN/PIK3CA mutations can occur concurrently in EC (3) ARID1A coexists with PTEN (4) FGFR and PTEN pathways may be interlinked. We suggest NGS be employed frequently in patients with endometrial cancer to identify targetable mutations. Additional larger studies are needed to characterize the interplay between mutations.

## Introduction

Endometrial cancer (EC) is the most common gynecologic malignancy in the western world and the second most common malignancy in women after breast cancer (1). It has an estimated incidence of 19.5 cases for 100,000 women and a mortality rate of 2.1 for 100,000 women (2). The incidence of EC is only expected to rise in the near future (3).

Although, the etiology of endometrial carcinoma is multi-factorial it can be further divided into modifiable and non-modifiable risk factors. Modifiable risk factors include nulliparity, obesity, physical inactivity, diabetes, hypertension and hormone replacement therapy (HRT) (2). Non-modifiable risk factors are age, race and genetic predisposition (4). Individuals who have a first-degree relative with endometrial cancer were found to have an increased risk of the disease, with a relative risk (RR) of 1.8 (1.7–2.0) estimated from a meta-analysis of 16 different studies of varying design and age range (5). Standard therapy for EC consists of total hysterectomy with bilateral salpingoophorectomy and lymph node assessment followed by adjuvant radio- and/or chemotherapy depending on final tumor characteristics and stage (6). Hormonal therapy is an alternative treatment for patients who wish to preserve their fertility, and for those with recurrent or metastatic disease without curative options (6, 7). Although the prognosis remains good for patients diagnosed with early-stage disease, for those diagnosed with recurrent or metastatic disease, options have been limited, and prognosis is poor.

Historically, two types of EC have been classified which was proposed by Bokhman in 1983, as Type I or Type II, based on tumor histology and presumed carcinogenesis (7–11). Type I Endometrioid EC (EEC) represents 80% of EC cases; most EECs are caused by an excess estrogen exposure that, in the absence of counteractive effects of progesterone, induces endometrial proliferation and subsequent endometrial hyperplasia and cancer (9). Type II Non-endometrioid EC (NEEC) is responsible for the remaining 20% of EC incidence and is assumed to develop independent of estrogen (9). Classification by histology provides important prognostic information and is helpful in identifying appropriate surgical and adjuvant therapy. A recent classification based on genomic-based molecular classification has been identified to assist in achieving optimal patient selection for adjuvant treatment in the management of EC, which is the comprehensive genomic analysis by The Cancer Genome Atlas (TCGA) (11, 12). The TCGA divides EC into four molecular subgroups based on mutational burden and copy number alterations (12). The four molecular categories are Polymerase epsilon (*POLE*) ultra-mutated, microsatellite instability hypermutated (MSI-H), copy-number low (CN-L), and copy-number high (CN-H) (11, 12).

It is promising that cancer genomic data may help us develop targeted treatment options against molecularly matched alterations that can be more effective and less toxic than traditional chemotherapeutic regimens. In 2018 the U.S. Food and Drug Administration (FDA) approved the first comprehensive genomic profiling (CGP) assay FoundationOne CDx™, a laboratory test designed to detect genetic mutations in solid tumors (13). In the field of cancer research, the use of next-generation sequencing (NGS) has increased in the last decade. NGS is a versatile technology for determining the sequence of DNA or RNA to study genetic variation associated with diseases or other biological phenomena. It is a sequencing process which involves fragmenting DNA/RNA into multiple pieces, adding adapters, sequencing the libraries, and reassembling them to form a genomic sequence (14). In 2005, NGS was introduced for commercial use and was initially called “massively-parallel sequencing,” because it enabled the sequencing of many DNA strands simultaneously, instead of one at a time as with traditional Sanger sequencing by capillary electrophoresis (15). This improved speed and accuracy, while reducing the cost of sequencing (15). NGS enables various applications including whole genome sequencing, whole exome sequencing, targeted resequencing, analysis of coding and non-coding RNA expression, alternative splicing and discovery of novel non-coding RNAs (11). Sequencing of mutations in diverse cancers shows the potential of discovering new diagnostic, prognostic, therapeutic, or predictive mutational statuses (16). There are few studies that explore the relationship between NGS molecular signatures and prognosis of EC. This study is an initial assessment of any common genomic alterations in recurrent or metastatic endometrial cancer on endocrine therapy that may aid in treatment paradigm.

## Materials and methods

We selectively chose six patients with established Stage IV disease who had at least 1.5 years of stable disease (SD) and ongoing benefit from hormonal therapy. They were treated in our clinic between the years 2016-2023. These patients were selected based on known benefit of at least 1.5 years on hormonal therapy, and exceeded the overall survival benefit of 10.2-14.7 months of endocrine therapy for EC reported in literature (17). Four patients received oral anastrozole at 1mg daily, 1 patient received oral megestrol 40mg twice daily, alternating with oral tamoxifen 20mg twice daily, and 1 patient initially received megestrol/tamoxifen but was switched to anastrozole when her disease progressed. Stage IV disease was defined as metastatic disease, according to the American Joint Committee on Cancer (AJCC) Tumor-Node-Metastases (TNM) system and the International Federation of Gynecology and Obstetrics (FIGO) staging system (18). Treatment response was assessed by either computerized tomography (CT) of the chest, abdomen and pelvis with intravenous (IV) contrast, or positron emission tomography (PET) scan performed every 3 to 6 months. We reviewed next-generation sequencing (NGS) on all six patients to assess if there were any common genomic or biomarker findings, other than the hormone receptor positivity, which could predict prolonged or sustained response to hormone therapy of ≥1.5 year (**Table 1**). Institutional Review Board (IRB) approval was obtained from Staten Island University Hospital and Northwell Health, New York.

**Table 1:** Next generation sequencing data was obtained for all six patients with endometrial cancer, who had achieved stable diseae (SD) for ≥1.5 year.

	Age (Years)	Hormone Receptor Status	Next-Generation Sequencing Data	PDL1 Status	Stable Disease (SD)
Patient 1	60	ER +/PR +	PIK3CA G118D ARID1A N1502fs*5 CTNNB1 D32Y PIK3R1 F456_Q457del PTEN R130G, K342*	PDL1 0%	5 years
Patient 2	90	ER +/PR +	ARID1A S881fs*10 FGFR2 C382R PIK3R1 R639fs*15 PTEN Y88* PTEN splice site 1651_166GGT>A SPOP E50K	PDL1 0%	1.5 years
Patient 3	69	ER +/PR +	ARID1A Q412* ASXL1 G181R ESR1 Y537N FGFR2 C382R PIK3CA Q546R PTEN T319fs*6	not available	5.5 years
Patient 4	66	ER +/PR +	ESR1 amplification MED12 G44V PIK3CA L113del TP53 R342*	not available	1.5 years
Patient 5	45	ER +/PR +	DNMT3A R882H FGFR2 S252W PIK3CA R88Q PIK3CA C604R PIK3CA H1047Y PTEN splice site 493-1G>T PTEN Q171K TP53 H178P	PDL1 <1%	2.5 years
Patient 6	75	ER +	ARID1A S11fs*89 ARID1A M50fs*59 ARID1A Q2176fs*48 BCOR N1425S CTCF Q198* CTCF T204fs*26 DNMT3A S714C FGFR2 Y375C FLCN H429fs*39 JAK1 K860fs*16 KDM5A R1261W KEL S502* MAP3K1 S398* MSH3 K383fs*32 MSH6 F1088fs*5 NF1 Q1987* PIK3C2B R287fs*92 PIK3R1 splice site 1300-1_1357del59 PIK3R1 I571fs*31 PTEN splice site 1027-2A>G RB1 R445* RNF43 G659fs*41 SPOP F125L TP53 N239S	PDL1 <1%	2.5 years

## Results

We reviewed the NGS results of six patients with stage IV endometrial cancer who are currently being treated with hormonal therapy in our clinic. Patients’ ages ranged from 60-89 years of age. Hormone receptor status was positive in the pathology of all six patients. Five patients were positive for both estrogen receptor and progesterone receptor (ER+/PR+), and one patient harbored only ER+. Programmed Death Ligand-1 (PDL-1) status was low in four patients and was not available for two patients. No patient in this series received PDL-1 targeted immunotherapy.

Review of next generation sequencing data failed to reveal one common genetic marker that could predict the stable disease (SD) benefit of ≥ 1.5 years observed in these patients. PTEN alterations were found in approximately 83% of patients. PIK3CA and PIK3R1 mutations were found in 67% and 50% of patients, respectively. ARID1A mutations were observed in approximately 67% of patients. TP53 and FGFR2 mutations were both found in 50% of patients.

Disease stabilization (SD), defined as no evidence of progression of metastatic lesions, or as regression of existing lesions on CT or PET imaging for at least 1.5 years, was achieved in all six patients. 5 patients continue to be followed in our clinic today, at the time of publication. 1 patient died after developing leukemia, but fulfilled the criteria of ≥ 1.5 years stable disease following hormone therapy.

## Discussion

NGS provides insight into the molecular profile of tumors, revealing possible targets for therapy. Improving patient survival hinges on improved understanding of molecular signatures of endometrial cancers and identifying predictors of treatment susceptibility. Although extensive tumor markers have been identified within endometrial cancer, the link to treatment response and prognosis is not as established as some other cancers such as breast or colon (19).

As mentioned, the traditional Bokhman classification of endometrial carcinoma, Type I EC has typically carried a better prognosis, and includes the endometroid subtype (10, 11, 20, 21). The survival benefit can likely be attributed to the low grade, well differentiated histology found in Type 1 EC (20). Type 2 EC has traditionally encompassed more high-grade subtypes, including clear cell and serous papillary endometrial cancer (11, 20). The Bokhman classification system has largely been replaced by NGS, in an effort to target therapies to the genetic profile of a patient’s cancer.

The TCGA method of classification can also be used to assess prognosis. Patients in the first (POLE ultramutated) group have exhibited good prognosis and progression-free survival, possibly from the endometrioid histology found in this category (11). Patients falling in the second category (MSI Hypermutated) have intermediate outcomes, but also have predominantly endometroid features (11). Patients in the MSI Hypermutated group also tend to harbor mutations in DNA mismatch repair (MMR) (11). Patients in the third (Low copy-number) group also have intermediate prognosis (11). They also exhibit endometroid features, and also tend to have overall severe changes in DNA or chromosomal quantity (11). Finally, patients in the fourth (High copy-number) group carry the worse prognosis, and encompass serous histology (11).

Hormone Receptor positivity has been established as a positive prognostic indicator in endometrial cancer (7, 21, 22). All six patients in our series exhibited positive hormone receptor status (5 had ER+/PR+, and 1 was ER+ only), and all subjects received endocrine therapy. Importantly, disease stabilization was achieved in all six patients, and has been maintained on hormone therapy for 1.5 to 5 years. At the time of this publication, all patients are still alive and being monitored in our clinic. Our series reinforces ER+/PR+ hormone receptor positivity as a favorable prognostic indicator in endometrial cancer. A large meta-analysis by Zhang et al. also established HER2+ as a poor prognostic indicator in EC, and it would be interesting to compare the survival of patients in our series with those that may be ER+/PR+/HER2+ on hormone therapy (21).

PTEN is one of the most common mutations found in endometrial cancer and acts as a tumor suppressor by cleaving the D3-phosphate of second messenger molecule PIP3 (23). PI3K (which acts under the influence of the *PIK3CA* Gene) is a molecule located in the intracellular bilayer membrane and is involved in activation of second messenger molecule PIP3 (24). High concentrations of PIP3 phosphorylate the protein AKT, whose downstream effect is cell survival via blockage of cytochrome c release from mitochondria (23). Mutations in the tumor suppressor PTEN and activating mutations of PIK3CA can overdrive this pathway (23, 24). Traditionally, PTEN and PIK3CA mutations have been thought to be mutually exclusive, when studied in lymphomas, breast and brain cancers (24–26). Despite this, it has been suggested that coexistence of both PTEN and PIK3CA mutations in endometrial cancer can carry a synergistic effect, resulting in overactivation of the same downstream pathway (27). Oda et al. have shown the co-existence of PTEN and PIK3CA mutations in endometrial cancers, and these results are reflected by the NGS data in our series: Of the 5 patients in this series who had PTEN mutations, 3 co-exhibited PIK3CA mutations (60%) (27). This is consistent with the data reported in a study that demonstrated PIK3CA mutations occurring more frequently in tumors that contained PTEN mutations (46% of tumors) (27). This is a finding unique to endometrial cancers, as numerous other studies of blood, brain, and breast cancers have deemed PIK3CA and PTEN mutations to be mutually exclusive (24–26). While it may seem that two mutations in the same pathway could carry a poor prognosis, all patients in this series who co-exhibited PIK3CA/PTEN were also hormone receptor positive (+), and went on to achieve at least 1.5 years of stable disease. Prior studies of these two markers have largely been *in vitro*. Based on the stable disease exhibited by the patients in this series, further clinical studies would be warranted to question the double mutation PIK3CA/PTEN as a prognostic indicator of disease progression. Further clinical studies would be able to characterize the prognostic benefit of PTEN loss alone, versus a coexisting PTEN/PIK3CA double mutation. In our study, the 2 patients who had a PTEN mutation alone also achieved at least 1.5 years of stable disease, in addition to the 3 patients with co-existing mutations. Several clinical trials assessing the activity of PIK3 inhibitors and AKT inhibitors in endometrial cancer have been done, however current data is inconclusive or limited by toxicity profiles and would warrant further trials (28).

Further downstream, the second messenger PIP3 acts on AKT/mTOR, a pathway involved in the regulation of the cell cycle, proliferation, and growth (29). Aberrancies in mTOR can influence tumor growth in many ways, from promotion of anabolism to evasion from natural killer (NK) cell immunosurveillance (29). Thus, mTOR has been an attractive target for therapy. A Cochrane review found that first-line treatment regimens for EC which had mTOR inhibitors had poorer progression free survival (PFS) when compared to treatment with chemotherapy and bevacizumab (30). However, when mTOR inhibitor monotherapy was used as second or third line treatment, there was some improvement in PFS in comparison to chemotherapy or hormone therapy (30). Although mTOR inhibitors alone are not associated with progression free survival, mTOR inhibitors co-administered with both hormone therapies and metformin have been shown to have clinical advantage in up to 50% of patients in a Phase II study (31). The study also found the highest clinical benefit in patients that were PR+ (31). The favorable presence of hormone receptor status in these patients is reflected in our series. Based on the Phase II study by Soliman et al, it would be interesting to study the addition of metformin and an mTOR inhibitor as part of the treatment regimen in these patients (31). Importantly, hormone receptors and PIK3CA/AKT pathway may not be independent of each other. Estrogen receptor activation affects AKT further downstream, and progesterone signaling via PI3K/mTOR/AKT (7). Further studies with larger patient population are vital to further characterizing the interplay of these targets, and whether mutations in these various targets could predict treatment susceptibility.

Mutations of the ARID1A gene are associated with altered subunits in the SWitch/Sucrose Nonfermentable Complex (SWI/SNF), a protein involved in chromatin remodeling, and largely acts as a tumor suppressor (32). ARID1A mutations coexist with PIK3CA or PTEN (32–34). In fact, ARID1A relies on these mutations for tumor genesis, and mouse models have shown that ARID1A mutations alone, without PTEN, cannot drive cancer formation alone (34). There is little data on ARID1A as a prognostic indicator for EC, likely because loss of the gene occurs very early in the tumor formation process (35). Werner et al. have demonstrated that loss of the gene was also associated with EC at an earlier median age (<66y), as well as a positive correlation with increased myometrial invasion and lower class of differentiation (35). Further studies would be needed to study ARID1A loss as a prognostic indicator, and whether there is any cross talk with the ER+/PR+ signaling, thus affecting response to hormonal therapy.

In our study, ARID1A mutations were found in four of the six patients. Consistent with literature, PTEN mutations were coexistent in all four of the ARID1A aberrant cases. This further reinforces prior studies suggesting that ARID1A alone is not sufficient for carcinogenesis (34). PIK3CA coexisted with only two of the four ARID1A mutant cases, and it would be interesting to further study the codependency of these two genes in tumor formation. Larger studies of NGS in EC patients are needed to determine if various combinations of mutations in these genes could predict response to hormonal treatment.

The final markers assessed in this study were FGFR2 mutations, present in four of the six patients (3 of whom are ER+/PR+, 1 only ER+). FGFR2 gene amplification mutations are prevalent in uterine cancers, and particularly in the endometroid histological type (36). FGFR2 mutations often present simultaneously with PTEN mutations, in about 77% of primary endometrial cancers (36). Our study was consistent with this data, with all four patients who had FGFR2 mutations also harboring PTEN alterations. The pathways are further interlinked, and *in vitro* studies have shown that PTEN aberrancy can impact response to FGFR targeted therapies (36). Further studies would be warranted to study this codependent relationship, and whether NGS revealing dual mutations in PTEN and FGFR2 can predict response to treatment.

Utilizing TCGA data as a prognostic indicator is a stepping stone to personalized treatment of EC. However the future of precision medicine goes beyond NGS. Non-Coding RNA (ncRNA) has recently been suggested as a prognostic indicator of EC, and as a marker of disease progression (11). ncRNA interact with mRNAs, thus silencing tumor suppressors or allowing expression of oncogenes (11). When used along with NGS data, ncRNAs have the potential to provide specific prognostic information, tailored to each patient’s disease (11).

The primary limitation of this brief research report is that the small sample size restricts the application of our conclusions to the general population. Another limitation is that we do not distinguish between types of hormone treatment in this study. While the majority of patients received an aromatase inhibitor, 2 patients received megestrol or tamoxifen during the course of treatment. Additionally, patients on medroxyprogesterone acetate (MPA) were not included in this study, since providers in our practice rarely use MPA due to adverse effects such as weight gain (7). Finally, we did not include NGS data from patients who did not survive EC. Future studies which include NGS from patients who died from EC could improve study validity.

## Conclusions

NGS in our patients did not identify any common genomic or biochemical marker that could be used in clinical practice to predict sustained response to hormonal therapy, partly due to small sample size. Notably, our NGS data does reflect the prevalence of various mutations reported in literature, with all patients achieving a stable disease state of at least 1.5 years. Further studies of a larger patient population with sustained response to hormonal therapy can provide insight into any one marker, or any set of combination of markers, that may predict benefit from hormonal therapy.

The Next Generation Sequencing data of the 6 patients in our case series reflects what is reported in literature: (1) Hormone Receptor (ER+/PR+) status can be targeted with endocrine therapies and be a positive prognostic indicator in achieving progression-free disease (7, 21, 22). (2) PTEN/PIK3CA mutations can occur concurrently in EC and target the same pathway (27). While this was reflected in our study, double-mutation patients also achieved stable disease (3) ARID1A is a tumor suppressor gene that must coexist with PTEN for malignancy (32–34). (4) FGFR and PTEN pathways may be interlinked and require further studies with larger sample size to identify any prognostic benefit (36). We propose that NGS be employed more frequently in patients with endometrial cancer to identify targetable mutations. Although this study focuses on 6 patients identified at our outpatient clinic with positive outcomes to hormonal therapy, we hope to conduct a thorough retrospective study to further study patterns in next generation sequencing for any correlation with survival.

## Data availability statement

The original contributions presented in the study are included in the article/supplementary material. Further inquiries can be directed to the corresponding author.

## Ethics statement

The Institutional Review Board (IRB) approval was obtained from Staten Island University Hospital and Northwell Health, New York. Written informed consent for participation was not required for this study in accordance with the national legislation and the institutional requirements.

## Author contributions

MD was the PI for this project, and initially came up with the idea for this case series based on her clinic patients. DA assisted in chart review, and procuring all the next generation sequencing information from the patients’ medical records. DC compiled all the data, and came up with the parameters and methods for this study. AR analyzed the NGS data, drew the conclusions, and did the research for all the discussions. All authors contributed to the article and approved the submitted version. 
